# Editorial for the Special Issue on Corrosion and Etching at Micro/Nanoscale

**DOI:** 10.3390/mi14020425

**Published:** 2023-02-10

**Authors:** Giorgio Luciano, Małgorzata Norek

**Affiliations:** 1 Institute for Chemical Technologies, National Research Council of Italy, Genoa Branch, 16149 Genova, Italy; 2 Institute of Materials Science & Engineering, Faculty of Advanced Technology & Chemistry, Military University of Technology, Str. Gen. S. Kaliskiego 2, 00-908 Warsaw, Poland

Micro- and nanoscale corrosion and etching at are important in several fields, from the fabrication of sensors and membranes to investigations of the properties of micro- and nanocomposites. The study of these phenomena is essential to acquiring knowledge on the physical and mechanical properties of synthesized materials and their resistance to corrosion.

Thus, in this Special Issue, we intend to present reviews and discussions of the theoretical and practical aspects of these processes. Four original research papers and one review article are published. One article [1] and the review [2] focus on soil corrosion, while the other articles focus on the design of photonic crystals [3], the study of a new microetching model in the field of oil and gas exploration [4], and the corrosion resistance of an amorphous coating [5].

Song et al. [1]. reported on the corrosion of galvanized steel used as a typical grounding grid material, whereby corroded samples were characterized and compared under different current conditions. Their results showed that the corrosion degree of galvanized steel was gradually aggravated with increasing current, and the corrosion degree of galvanized steel under a DC current was greater than that under an AC current.

Zhang et al. [2] reviewed soil corrosion in grounding grids and provided details of its causes, mechanisms, types, and influencing factors, as well as the corresponding detection technology and protective measures. Moreover, the paper pointed out the impact that soil corrosion can have on the grounding grid system. Topics such as the impact mechanism of an AC stray current, new corrosion detection technology, and better protective measures were recommended for in-depth study in the future.

Pligovka et al. [3] developed three types of niobia nanostructured films on glass substrate via porous alumina-assisted two-step anodizing and chemical post-processing. They investigated the anodic behaviors, morphology, and optical properties of the films, highlighting how anodization is used in developing composite and functional materials for electronic devices, various sensors, and other optically active materials, including metamaterials, materials for solar cells and solar fuel production, and other energy storage applications.

Wu et al. [4] studied the microscopic mechanism of formation damage caused by drilling fluid, focusing on the microscopic mechanism of fluid damage. For this purpose, they designed a new microetching model (MEM), along with displacement equipment. The pore networks of rock samples were extracted from thin-section images and etched onto a thin aluminum sheet using a laser, and oil-based drilling fluid was used to displace the stratum water in the MEM. A core flooding experiment, permeability measurement, and SEM observations were performed, which showed that, for low-porosity and low-permeability sandstone, the main forms of formation damage caused by drilling fluid include solid and liquid damage.

Chu et al. [5] studied the corrosion resistance of an amorphous coating and composite coatings with various proportions of AT13 (Al_2_O_3_–13 wt.% TiO_2_) ceramic as additions to 3.5 wt.% NaCl solution. They confirmed that the addition of second-phase content enhanced the electrochemical corrosion performance and that when AT13 was 15 wt.%, the coating composite had the lowest corrosion current density and the highest corrosion potential.

The guest editors would like to thank all the authors who contributed to this Special Issue, the reviewers for their constructive evaluation of the manuscripts, and the publisher for their kind and efficient cooperation. We are pleased to introduce the collected articles to readers who are interested in the phenomena that occur during micro/nanoscale corrosion and etching.
